# Draft Genome Sequence of *Escherichia coli* Strain ATCC 23506 (Serovar O10:K5:H4)

**DOI:** 10.1128/genomeA.00049-13

**Published:** 2013-02-28

**Authors:** Brady F. Cress, Zachary R. Greene, Robert J. Linhardt, Mattheos A. G. Koffas

**Affiliations:** a Department of Chemical and Biological Engineering, Center for Biotechnology and Interdisciplinary Studies, Rensselaer Polytechnic Institute, Troy, New York, USA; b Department of Chemistry, Chemical Biology, Center for Biotechnology and Interdisciplinary Studies, Rensselaer Polytechnic Institute, Troy, New York, USA; c Department of Biology, Center for Biotechnology and Interdisciplinary Studies, Rensselaer Polytechnic Institute, Troy, New York, USA

## Abstract

We report the 5.101-Mbp high-quality draft assembly of the *Escherichia coli* strain ATCC 23506 (serovar O10:K5:H4, also known as NCDC Bi 8337-41) genome. This uropathogenic strain, commonly referred to as *E. coli* K5, produces *N*-acetyl heparosan, a glycosaminoglycan-like capsular polysaccharide and precursor to the anticoagulant pharmaceutical heparin. Metabolic reconstruction of this genome will enable the prediction of gene deletions and overexpressions that lead to increased heparosan production.

## GENOME ANNOUNCEMENT

*Escherichia coli* is the most well-characterized organism commonly utilized in metabolic engineering research. As metabolic engineers seek to further increase the innate production capacities of unique *E. coli* strains, it is valuable to develop genome-scale reconstructions (GSR) of metabolism in order to accurately predict genetic engineering interventions that lead to an improved phenotype. The K5 capsule is composed of *N*-acetyl heparosan, a group II capsular polysaccharide (CPS) consisting of a repeating [→4) β-d-glucuronic acid (GlcA) (1→4) *N*-acetyl-α-d-glucosamine (GlcNAc) (1→]_*n*_ disaccharide unit (1). Although the gene cluster encoding the enzymes required for the biosynthesis of K5 CPS has been characterized elsewhere, annotation of the whole genome sequence will lend further insight into the molecular mechanisms of capsular polysaccharide biosynthesis and transport. The characterization of all genes involved in lipopolysaccharide (LPS) biosynthesis will also enhance understanding of CPS-LPS interactions, while comparative genomic studies between this uropathogenic *E. coli* (UPEC) strain and nonpathogenic strains might identify the virulence factors required for infection of the urinary tract.

Genomic DNA was purified from *E. coli* strain ATCC 23506 with an Invitrogen PureLink Genomic DNA mini kit. The genome was sequenced using the Illumina HiSeq 2000 sequencing system, which produced 104 M paired-end reads of 101 bp, with an insert size of 400 bp. Approximately 28M random reads were assembled with Velvet v1.2.07 (2) at an optimal hash length of 93. The final genome assembly has approximately 38-fold coverage and contains 190 supercontigs composed of 224 contigs (>200 bp in length) with a total size of 5,101,025 bp, an N_50_ contig length of 129,677 nucleotides, and a mean G+C content of 50.6%. Assembly data were deposited in the EMBL nucleotide sequence database.

The draft genome was annotated by the Rapid Annotations using Subsystems Technology (RAST) server (3) using Glimmer3 as a gene caller (4), which predicted 5,030 coding sequences (CDSs) with an average length of 880 bp (3,815 CDSs have functional predictions), 86 tRNA-encoding genes, and 25 rRNA-encoding genes. RAST was also used to construct a draft metabolic model (5) containing 1,156 genes, corresponding to 1,408 reactions with 1,112 metabolites (including 4 gap-filling reactions and an artificial biomass reaction).

Of particular interest, the sigma factor *rpoF* (gene *fliA*)—required for upregulation of the flagellar regulon—was absent from the genome, along with several other flagellar biosynthetic genes; a motility assay confirmed that uropathogenic *E. coli* strain ATCC 23506 is nonmotile in soft tryptone agar (data not shown), a result consistent with those of a previous investigation of an *E. coli fliA* deletion mutant (6). A detailed comparative genomics study is under way between this strain and other recently sequenced strains that also produce glycosaminoglycan-like capsular polysaccharides of pharmaceutical and nutraceutical relevance. Such analyses will improve the understanding of CPS biosynthesis regulation and the effect of the metabolic landscape on CPS production in pathogenic strains that depend upon the capsule as a “molecular camouflage” for host colonization.

### Nucleotide sequence accession numbers.

The annotated draft genome sequence was deposited in DDBJ/EMBL/GenBank under the accession no. CAPK00000000. The version described in this paper is the first version, CAPK01000000.
